# “We don’t need no education” – a qualitative study of barriers to continuous medical education among Danish general practitioners

**DOI:** 10.1186/s12909-023-04432-9

**Published:** 2023-06-19

**Authors:** Helle Ibsen, Gunver Lillevang, Jens Søndergaard, Niels Kristian Kjaer

**Affiliations:** 1 Department of Public Health, Research Unit of General Practice, University of Southern Denmark, Finsensvej 35, 6700 Esbjerg, Denmark; 2 Department of Clinical Medicine, Faculty of Health and Medical Sciences, University of Copenhagen, Blegdamsvej 3B, 2200 Copenhagen N, Denmark; 3 Department of Public Health, Research Unit of General Practice, University of Southern Denmark, WP 9, 5000 Odense, Denmark

**Keywords:** Barriers, Continuous medical education, General practice, Qualitative study

## Abstract

**Background:**

Continuous medical education is essential for the individual patient care, the society, and the wellbeing of the general practitioner. There has been research into the reasons for participation in continuous medical education, but little is known about the barriers to participation. To tailor continuous medical education targeting general practitioners who are currently deselecting education, systematic knowledge of the barriers is needed.

Continuous medical education can in addition to professional growth stimulate job satisfaction, diminish burnout, and reinforce feelings of competence. Continuous medical education may have positive implications for patients and for healthcare expenditures.

Despite renumeration and a comprehensive continuous education model some Danish general practitioners do not participate in continuous medical education.

**Methods:**

From a total of 3440 Danish general practitioners 243 did not apply for reimbursement for accredited continuous medical education in a two-year period. Ten general practitioners were selected for an interview regarding maximum variation in practice form, number of listed patients, seniority as a general practitioner, geography, gender, and age. All ten selected general practitioners accepted to be interviewed. The interviews were analysed using Systematic Text Condensation.

**Results:**

Each of the ten interviewed general practitioners mentioned several barriers for participating in continuous education. The barriers fell into three main categories:

- barriers related to the individual general practitioner
- barriers related to the clinic
- barriers related to the accredited continuous medical education offered

**Conclusions:**

Approximately 7% of the Danish general practitioners did not participate in accredited remunerated continuous medical education. A knowledge of the barriers for participating in accredited continuous medical education can be used to better target continuous medical education to the general practitioners.

**Supplementary Information:**

The online version contains supplementary material available at 10.1186/s12909-023-04432-9.

## Introduction

Continuous medical education (CME) is essential for both the individual patient care, the society, and the wellbeing of the general practitioner (GP) [1–3]. While there has been research into the reasons for participation in CME, little is known about GPs’ barriers to participation in CME. The public has a legitimate interest in GPs’ CME, so nonparticipation deserves our attention. In order to ease non-participators access to CME we require knowledge of the barriers. Knowledge which allows educators to tailor meaningful CME to GPs who are currently deselecting CME.

## Background

### CME as expertise development and improvement of patient care

While all GP trainees acquire new knowledge through their specialist training, their post-training continuing medical education has received little attention [4]. Consequently, the World Organisation of Family Doctors (WONCA) has developed global standards in CME to assist general practitioners in providing excellent patient care [4].

WONCA’s initiative is important because when a professional achieves an acceptable level of skills, more experience does not, by itself, necessarily lead to improvements [5]. Ericsson describes deliberate practice as a prerequisite for expertise development and higher quality in patient care: Significant improvements in performance have been seen when professionals have a well-defined goal, motivation to improve, and are provided with feedback and ample opportunities for repetition and gradual refinements [5]. Learning from and with colleagues is an important source of both new information and strategies for applying that information to practice [6].

A comprehensive CME programme for general practitioners is essential for developing and maintaining high professional standards in general practice, especially if it supports both professional development and professional motivation [7].

### CME benefits patients, GPs and society

GPs who do not participate in practice based small group learning (PBSGL) have an increased risk of burnout [8]. Although there is not shown a causal link between participating in PBSGL and less burnout, non-participation in CME may be seen as a warning sign [8].

Danish GPs state that avoiding burnout is an important reason for participation in CME [2]. CME can stimulate job satisfaction, diminish burnout, and delayed retirement is seen among GPs who thrive in their job [2, 9]. Furthermore, participation in CME can reduce feelings of professional isolation and reinforce feelings of competence and autonomy in physicians [10].

To be continuously competent in delivery of high-quality health care is important for being a GP and is the most important motivational factor for participating in CME [1, 11]. Especially if the learning activities are related to patient care relevant for the GP’s daily work. GPs are less motivated by overall competence improvement goals [12]. Educational research emphasizes the importance of individual needs analysis and self-assessment [13, 14] but also the importance of providing strategies on how to implement new knowledge into daily clinical practice [2, 15].

In 2014 Kjaer, Steenstrup et al. found that Danish GPs used many different types of CME activities including online updating of knowledge and problem based small group learning (PBSGL) [2]. Danish GPs chose their CME activities based on personal needs analysis related to patient problems, a need to be professionally updated, and a need to meet engaged colleagues [2]. Furthermore, CME activities were used to prevent professional fatigue and burnout [2].

Participating in CME is essential for the individual patient’s health care and for well-being of the GP. Several studies indicate that GPs’ work conditions and mental well-being may have positive implications for their patients and for healthcare expenditures [3, 16].

CME activities show promise as a strategy to recruit and retain physicians in less attractive specialties [10].

Society has a legal interest in the GPs continuous medical education. Valuable CME should therefore address both the needs of individual clinicians, the populations they serve, and the organisations within which they work [17].

### Barriers for participation in CME

A CME report from the Danish Association of General Practitioners (PLO) from 2018 shows that—seen over a year—approximately 20% of the Danish GPs do not apply for financial reimbursement of CME activities [18]. Our aim of the study is to uncover which reasons Danish GPs state as barriers for attending accredited remunerated CME activities.

Little is known about barriers for participation in CME among GPs. In Portugal, lack of time as well as bureaucracy overload has been pointed out as the main barrier for implementing a digital CME platform [19]. The lack of time led to a feeling of lack of discipline, laziness, and guilt [19].

A previous study on barriers towards mandatory CME among doctors in Ireland identified five main themes related to participating in CME [20]. Two themes (evidence of participation and a competence scheme) are not relevant in a Danish setting since we have an auto-filled electronic CME-log. Another two (workplace challenges and access to relevant CME) correspond to themes in our study. The last theme was that some Irish doctors consider mandatory CME as irrelevant and see it as an added stressor [20].

To overcome barriers against CME the Swedish Association of General Practice suggests that statements of recurring CME should be incorporated in contracts between health authorities and health care units. They have published a set of guidelines regarding Swedish GPs’s CME. They conclude that CME credits for certification purposes does not ensure that educational measures have been effective [21]. It is not known whether incorporation of CME in the GP’s contract can reduce the proportion of GPs who opt out of CME.

Our aim of the study is to uncover Danish GPs’ barriers for attending accredited remunerated CME activities. We will use this knowledge to construct a questionnaire to be sent to all Danish GPs.

## Methods

### Setting

The Danish GPs have a central and strong role in the Danish health care system both as gatekeepers and as responsible for most of the primary care [22]. GPs undergo six years of specified postgraduate training before being able to practice as a GP in Denmark. A description of the Danish System can be seen in Appendix 1.

### The current CME programme in Denmark

The Danish CME programme for GPs is based on professional integrity and trust without revalidation or recertification. The programme consists of a voluntary part based on the GP’s individual needs analysis and a mandatory part based on a mutual needs analysis of general practice as a profession. The mandatory CME uses the national curriculum of Danish family medicine as a framework and was developed involving GPs and other stakeholders [7]. The Danish CME programme is based on accredited activities renumerated by up to approximately € 6500 per year. There is no funding for non-accredited activities. The voluntary and mandatory CME is practically equally reimbursed. The GP can choose to use allocated funds from one year in the following year. According to the collective agreement between Danish Association of General Practitioners (PLO) and Danish Regions the GP has an obligation to develop and maintain his or her own competencies [22].

Despite the renumeration and a comprehensive CME model [7] not all Danish GPs participate in CME.

A CME report from PLO from 2018 shows that—seen over a year—approximately 20% of the Danish GPs do not apply for financial reimbursement of CME activities [18]. Principles for the Danish GPs’ CME programme is shown in detail in Appendix 2.

### Sampling

A total of 3440 General Practitioners were in 2016–2017 registered in the Danish Association of General Practitioners (PLO) [18]. The PLO’s CME department (PLO-E) had in the same two years (2016–2017) identified 243 GPs, who had not applied for their reimbursement for CME. This group formed the total data material for our study (*n* = 243) [18].

The Region of Southern Denmark represents an average region in Denmark when it comes to demographic, types of GP clinics, and distribution between rural and urban areas. In the Southern Region of Denmark, 44 GPs had not applied for reimbursement in 2016–2017 [18]. We selected ten GPs from the Region of Southern Denmark for individual interviews based on the assumption that ten interviews would provide us with sufficient qualitative data (for quantitative data on the 44 GPs see Appendix 3).

We applied a purposeful sampling strategy [23] aiming to obtain maximum variation regarding practice form, number of listed patients per GP, working years as a GP, geography (distance to university), gender, and age of the GP. Our hypothesis was that these characteristics might influence barriers for CME.

In our qualitative sampling we aimed to get a broad insight into the barriers and to gain a wide firsthand knowledge of GPs experiences with barriers for participating in CME [24]. Ten GPs from different practices in the Region of Southern Denmark were included Table 1.

**Table 1:** Characteristics of the ten included GPs

Respondents		
Gender	Female	4
	Male	6
GP age in years	≤ 47	3
	48–66	4
	> 67	3
Practice type	Singled handed	3
	Partnership	7
Number of full-time capacities	1	3
	2–3	4
	> 4	3
Number of patients/GP	< 1500	3
	1500–2000	4
	> 2000	3
Seniority as a GP in years	< 5	2
	6–15	4
	> 16	4
Distance to hospital in km	< 25	5
	> 25	5

We chose a face-to-face interview over focus group interviews to reduce the risk of informants being reluctant to participate due to the risk of appearing professionally out of date. Open-ended questions sent in advance were preferred over a more structured interview guide to reduce the risk of informants regretting their pledge to participate in an interview concerning non-participation in funded CME activities. In an attempt to stay as open-minded as possible we chose not to use a detailed interview guide [23]. If the GPs stated “lack of time” they were asked to give further information about the perception of lack of time.

The GPs included for interviews received an invitation by letter from PLO followed by a phone call from the first author (HI).

### Data collection

#### Qualitative data

All ten selected GPs accepted to be interviewed. A mail containing five open-ended questions inspired by Guided Self-determination [25] and two close-ended questions was sent to all informants 7 to 14 days before the interview (for questions sent in advance to informants see Appendix 4).

The interviews were conducted face-to-face in the informant's own clinic. In this way, the informant was at “home” and the interviewer was the “guest”. The interviewer (HI) is a GP herself which made the relation between the interviewer and informant as equal as possible thus allowing use of a common language. The interviewer was supervised by two co-authors (GL, NKK). If a face-to-face interview was not possible due to practical issues of the informant the interview was conducted by telephone, and the informant got the possibility to give written answers in addition to the telephone interview. The face-to-face interviews were digitally recorded with the consent of the informants.

#### Quantitative data

Data from the Danish CME reimbursement register for 2016 and 2017 was collected in anonymous form.

### Data analysis

#### Qualitative data

All ten interviews were transcribed verbatim by the interviewer (HI) and analysed using Systematic Text Condensation [23, 26]. Systematic Text Condensation involves four steps. First, the transcribed interviews were read thoroughly to get a total impression of the data material. Preliminary themes associated with the research question were generated and written down individually by three of the authors (GL, NKK, HI, all experienced GP with knowledge of qualitative research). The three researchers discussed their individually chosen themes (a total of seven themes) and agreed on three themes of importance to the research question.

Next, text fragments (meaning units [26]) representing meaningful aspects regarding the chosen themes was identified and sorted into three code groups. Through discussion the code groups were adjusted and refined. Each code group was further split into 2–4 subgroups.

Meaning units of each individual subgroup were compiled into one comprehensive artificial quotation, a condensate. Meaningful units that did not fit were either left out or placed in another subgroup.

Finally, based on the artificial quotations an analytic text for each code group was developed and the essence was expressed in separate category headings. All transcribed interviews were then reread in search for data that might challenge our final conclusions. Decisions on each step in the process of analysis were based on discussions between the authors.

#### Quantitative data

Data from the Danish CME reimbursement register informs how many GPs who do not apply for financial reimbursement per year. But since Danish GPs can use allocated funds in a time span of two years, we extended our data register period to two consecutive years.

## Results

### Quantitative results

**Fig. 1:**
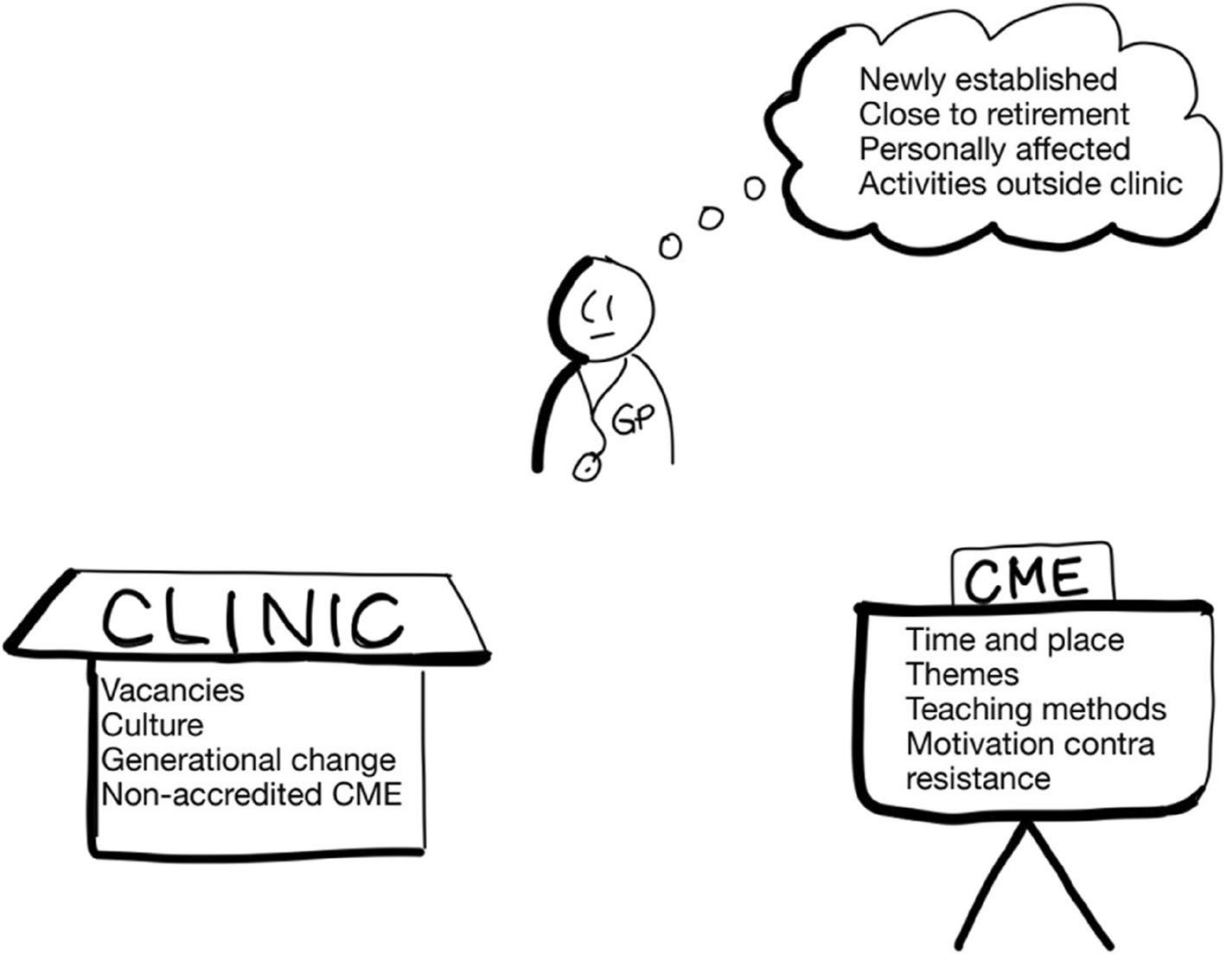
Barriers related to the GP, to the clinic, and to the CME

By looking at a two-year period (2016–2017) instead of one year, the proportion of GPs not applying for their allocated funds declined from 20% [18] to 7% (243 GPs/3440 GPs) Fig. 1.

### Qualitative results

Each of the ten interviewed GPs mentions several barriers for participating in CME. These fall into three main categories:

- barriers related to the individual GP
- barriers related to the GP’s clinic
- barriers related to the accredited CME offered

### Barriers related to the individual GP

The newly established GP and the GP close to retirement both indicate their position in working life as reasons for opting out of CME. The newly established GP argues that she is overwhelmed by tasks related to the start-up as a GP, tasks which was unknown for her as an employed doctor.

*“… and (I) ended up in a cauldron where I must do everything myself… I think my excuse is, I did not have the surplus. I must do VAT myself; I must report salary… things I was not used to do before … things that just had happened…” (Female, eight years’ experience as a GP).*

The GP close to retirement miss the social aspect of attending a course. Due to retirement of former friends and colleagues, she reports previous experiences of feeling alone on a course.

*“I will not say I stay at home because I expect that I will not meet anyone I know. But I take it into consideration when choosing a course… because there is also a social aspect to it…” (Female GP, 65 years).*

In the category related to the individual GP we also find the personally affected GPs; the GP with ongoing patient’s complaints, the GP who herself has health problems, or the GP with illness in close family.

*“…and I had to meet with the medical officer of the Danish Patient Safety Authority, and then suddenly there was a tight supervision on “little me”. I have never had a complaint before in my entire working life. So, I was kind of stressed…” (four years’ experience as a GP, partnership practice).*

GPs state professional activities outside the clinic as barriers for participation in CME. Both due to limited possibility to be absent from the clinic and because the GP meet his needs for medical education through other activities.

*“… I've been busy with everything else… been a teacher, a medical consultant and been the leader of some professional medical groups…. I have an agreement with my colleagues in this clinic concerning how many days I can be away… So, if there are many activities … but I learn a lot from being with these people, because they are good at what they do, aren’t they?” (45 years, partnership practice).*

### Barriers related to the GP’s clinic

Several GPs are struggling with lack of medical resources in their clinic due to vacancies, retirement of a GP without immediate replacement of a new colleague, or illness among colleagues. The GPs find it difficult or impossible to be absent from their clinics.

*"… the last few years I have not had time for anything. Not even this (the interview). I must be here on weekends to make ends meet. And then having to spend a day or two on this (CME) … and it is the same with going on holiday—I do not want to go on holiday anymore…” (Singled handed GP, 2600 listed patients).*

Some GPs try to manage by help from colleagues in nearby clinics. Others considered hiring a substitute but experienced challenges due to availability or salary demands. Especially the singled handed GPs find it financially problematic to hire a substitute.

*"I am the only singled handed GP in my area, so I have to beg to have my patients cared for while I am away. There are insufficient opportunities to get a substitute both for courses and for holidays, so consequently I only ask for help when it is most necessary. GP substitutes are insanely expensive, I will never even get anywhere near the salary a substitute should have” (Female, singled handed GP).*

Generational change is mentioned as another barrier related to the clinic. It can be due to lack of medical resources in a period around the generational change but even when a retired GP is directly replaced by a new GP, barriers to CME can occur.

*"… there is not much tradition for that (CME) in this house either, it is an “old” house. But now we try to prioritize it when we are getting younger—or relatively younger. There is a culture in a house that you can be able to change, but it requires a bit of work to change it. " (Female, eight years’ experience as a GP).*

Several of the interviewed GPs talk about educational activities taking place in their clinics. Activities which are not recognized as CME because of no application for remuneration or because of sponsored activity. Educational activities can be to invite an external specialist from the local hospital to teach lung diseases in the clinic or it can be to have a structure in the clinic concerning internal training.

*“… we also have an annual cycle where we go through the major diseases, we have diabetes once a year, there is asthma, there is COPD and hypertension and heart failure and things like that. In the clinic we have a culture where we teach each other when we have been on a course… "(Female, six GPs in partnership practice).*

*“We have an extensive educational culture, i.e., supervision culture in our practice. We have something we call a “professional club” where both GPs and trainees take turns teaching each other. We have a learning culture – we are not afraid to make mistakes, because we know we will. So, part of our staff meetings is about adverse events and dealing with conflicts.” (Male, four GPs in partnership practice).*

### Barriers related to the accredited CME activities offered

Factors related to form and content of the accredited CME offered can be barriers for participating. Some GPs request short and precise CME with emphasis on new knowledge. Some prefer cathedral teaching rather than group work.

*“I like that it (the CME) is concrete, with a minimum of wasted time. If you do not know anything, it does not lead to anything to sit and talk to someone who knows as little as you do. I want to get some facts served right away. “(Female, 19 years’ experience as a GP).*

Some of the GPs experiencing barriers to CME do not find courses covering themes that they prefer when searching for CME. The themes that some GPs are missing in the accredited CME can be specialised areas as maritime medicine or courses concerning treatment that is alternative to conventional medicine.

*"Not everything is accredited CME. I must pay for it myself. I have paid for my education for two years. I know that many do not like it (non-conventional medicine) or do not think it is supported by research. But there are many things we cannot support with research. I use it in my everyday life, and I know that it works." (GP four years’ experience, partnership practice).*

When it comes to courses held abroad the GPs disagree with each other. Some prefer courses held abroad, since being away from daily life helps them with concentrating on the course, exchanging experiences with colleagues, and getting new inspiration. Other GPs struggle to find time and energy to participate in a course that last several days.

Regarding arrangements in the afternoons or evenings, the interviewed GPs are more aligned: They generally lack accredited CME activities offered in their neighbourhood.

*"I don't mind moving to a course with overnight stay, whether it is Jutland or Zealand (the GP’s clinic is located on Funen). But half-day events that are in Jutland, it will be without me. If the course is one hour away, I will probably not come. If it is those “four- hours-something”, it must be close.” (Male, 56 years).*

Few GPs mention problems with insufficient provision of courses or difficulty in seeking remuneration after a course.

*"When I think I should participate in a course, the course is oversubscribed because I have not made my decision early enough, right?"*

One GP declares lack of motivation to participate in CME because the GP perceive the accredited CME offered as an attempt to push the GPs to be able to manage more than they are capable of. The GP sees a gap between the PLO-E and the everyday life of the GPs working in areas with a shortage of GPs.

*“It does not necessarily help attending a course in organizing my life in practice and how I become better at adapting to the pace of work. I think one must look at what is realistic to be able to do. I have not been on one of those courses. Just the headline makes me dislike the course and make me think: Well, do I have to—adapt? There is no doubt that you can obtain many useful tools to manage a stressful everyday life, but the tendency will just be that we pour more on the GPs.” (GP 47 year in a partnership practice).*

## Discussion

### Summary of main findings

Most of the interviewed GPs not participating in CME stated more than one barrier for participating in accredited CME. The reasons fell into three main categories of barriers:

- barriers related to the individual GP
- barriers related to the GP’s clinic
- barriers related to the accredited CME offered

### Hidden education behind the barriers?

In each of the above-mentioned categories there was hidden educational activities. In the category “Barriers related to the individual GP” we found GPs acting as teacher/course leader or course administrator.

Educational activities occurred in several of the GPs’ clinics but were not recognized as CME in our study due to no application for remuneration.

In the category “Barriers related to the CME” some GPs participated in accredited CME but found it too complicated to apply for remuneration or had simply forgotten to apply.

Some GPs found non-accredited CME from course providers who offered other themes. Nine of the ten informants participated in educational meetings arranged by the pharmacological industry. All nine spontaneous substantiated their statements with a further explanation. They explained their choices by geography (little distance to the professional meetings offered), or by educational form (didactic learning instead of peer engagement).

A challenge of this “hidden education” is that we do not know the quality and relevance since it has not passed any accreditation.

### Strengths and limitations

By using data from PLO-E we obtain data from 99% of the GPs in Denmark. Furthermore, it is a strength that all ten invited informants accepted the invitation.

Face-to-face interviews can both be seen as a strength and a limitation. The face-to-face interview can provide a basis for trustful intercollegiate conversation, but informants can also feel shy to reveal sensitive issues. However, during the interviews themes which we consider “difficult” when talking to an unknown interviewer were discussed. It was themes as patients’ complaints, personal crises, and collaboration problems in the clinic. It seems as although the informants were not anonymous in the conversation, they felt anonymous in the context.

The small sample size of ten GPs proposes a risk of inadequate coverage of the entire spectrum of barriers for participating in CME. However, after the first eight interviews no new barriers presented which could indicate data saturation.

In case of informants related practical issues, we allowed telephone interviews rather than deselecting informants to avoid selection bias.

### Comparison with existing literature

None of the interviewed Danish GPs expressed a feeling of lack of discipline or laziness. This contrasts a Portuguese study, where the GPs describe a feeling of lack of time which leads to a feeling of lack of discipline, laziness, and guilt [19]. It can be due to none of the Danish GPs having these feelings, or because we asked for the reason behind the feeling of lack of time which may soften a sense of guilt.

The barriers highlighted by GPs in our study are broadly in line with barriers found in recent studies. The three most reported barriers to CME among doctors are lack of time, cost, and location of CME activities [27]. As we explore barriers for participating in funded CME, cost is less relevant in our study due to the Danish reimbursement system [7].

In our study nine of ten informants participated in educational meetings offered by the pharmacological industry. These activities may fill a gap in the accredited CME offered, but there can be ethical and educational challenges. Educational meetings sponsored or organized by the industry often lack relevance or include a specialist who is not familiar with the circumstances of the daily work as a GP [28] and tend to have a focus on pharmacological solutions favoring a specific product rather than best practice for the patient [29].

The Danish CME programme consists of two parts. Centrally planned mandatory activities and self-chosen voluntary activities. In some countries a shift towards mandatory CME has resulted in more negative attitudes towards CME [20]. Macdougall et al. outlines pros and cons of mandatory CME versus unregulated self-directed CME, noting the feeling that mandatory CME may turn into “tick-box” exercises with more focus on requirement rather than learning [30].

More research is needed to uncover whether there is a difference between Danish GPs' attitudes towards centrally planned mandatory activities or self-chosen voluntary activities and if it has an impact on barriers to participation in CME.

### Implications for the GP, the patient, and the future CME

In our study a little less than 7% of Danish GPs deselect accredited renumerated CME despite fair renumeration. This figure may appear low, acknowledging GPs close to retirement and newly educated young GPs are among them. However, the Danish GPs not attending CME have more than 400.000 patients enlisted in their clinics. Since CME activities probably improve the quality of patient care and influence management of patients [31], CME organizers and health authorities should have focus on how to recruit the non-participants to meaningful CME even though we do not know the full extent of the “hidden education”. All ten interviewed GPs stated they want to participate in CME if they were able to overcome the experienced barriers.

Some identified barriers may easily be addressed by providers of CME. Others may be more difficult to surpass e.g., expressive workload. But we assume it is possible to surpass most of the barriers in deliberate collaboration between the GPs, their clinics, CME-providers, health authorities and GP research departments combining local and international findings and experiences. This process needs to approach the difficult balance between trust in professional integrity versus societal accountability [2, 32].

To develop appropriate solutions more research is required to determine the quantitative distribution and the significance of the detected barriers. In a newly launched national questionnaire survey, we will uncover the magnitude and significance of Danish GPs’s barriers for attending CME.

## Conclusions

Less than 7% of the Danish GPs do not participate in accredited remunerated CME. Nevertheless, it corresponds to 400.000 listed patients having a GP who does not participate in accredited CME. The barriers for participating in CME fall into three main categories: Barriers related to the individual GP, barriers related to the GP’s clinic and barriers related to the accredited CME offered. A knowledge of the barriers for participating in accredited CME can be used to better target CME for some of the GPs who are currently deselecting CME.

## Supplementary Information

**Additional file 1.**

## Data Availability

The dataset used and analysed during the current study is available in Danish from the corresponding author on reasonable request.

## Abbreviations

CME: Continuous medical education
GP: General practitioner
PBSGL: Problem based small group learning
PLO: Danish Association of General Practitioners in Denmark
PLO-E: Danish Association of General Practitioners’ CME department
WONCA: World Organisation of Family Doctors
