# Supplementary material for: “We don’t need no education” – a qualitative study of barriers to continuous medical education among Danish general practitioners
Source: BMC Med Educ. 2023 Jun 19;23:450. doi: 10.1186/s12909-023-04432-9 (PMC10278246; doi:10.1186/s12909-023-04432-9)
Supplement: Supplementary file 1 — Additional file 1. [file 12909_2023_4432_MOESM1_ESM.docx]

**Appendix 1:**

**The Danish health care system.**

The Danish general practitioners (GPs) have a central and strong role in the Danish health care system both as gatekeepers and as responsible for most of the primary care. Referral from a GP is required for most office-based specialists and always for in- and outpatient hospital treatment.

Danish primary healthcare is organised as a national public system providing free medical care. All citizens can choose to be listed in a GP-clinic. The average number of listed patients is 1600. GPs undergo 6 years of specified postgraduate training before being able to practice as a GP in Denmark. Doctors who complete the Danish GP training program have been recognised as medical specialist since 1993.

Danish GPs are self-employed, working on contract for the public funder; the Danish Regions (responsible for the national health service including the provision of general practice). The national agreement details not only services and reimbursement but also opening hours and required postgraduate education.

There are mainly two practice forms in Denmark: Partnership practices and single-handed practices. Both partnership and single-handed practices can choose to work in collaboration. Collaboration practices have their own listed patients and separate economy but typically share clinic buildings, clinic equipment and personnel. The income of a Danish GP is partly payment per listed patient and partly fees for services.

Helle Ibsen. Personal information 01.01.2023

**Appendix 2:**

**Principles for the continuous medical education programme for Danish General Practitioners**

The Danish GPs’ CME programme is based on the following principles:

- It consists of partly centrally planned activities and partly self-chosen activities
- There is no certification/revalidation
- It is based on accredited activities remunerated by up to approximately € 6500 per year
- There is no funding for non-accredited CME activities
- Individual GPs decide which of the accredited CME activities they wish to attend
- Three days each year are allocated to centrally planned CME activities
- Courses arranged by the pharmacological industry are not accredited
- Renumerated CME activities can be both practice-based small group learning and more traditional CME courses
- Activities such as reading journals and/or self-studies cannot be accredited
- 25% of the funds for self-chosen CME can be used on study material
- A two-year rule means that funds from one year can be used in the following year

**Appendix 3:**

The 44 GPs not attending CME in the Southern Region of Denmark/All GPs in Denmark

|  | **44 GPs not attending CME (n= 44)** | **All GPs in Denmark (n 3440)** |
| --- | --- | --- |
| **Male/female ratio** | 28/16 (1,75) | 1725/1718 (1,00) |
| **Average Age years (variation)** | 50 years (39-74) | 53 years (34-85) |
| **Singled handed/partnership practice ratio** | 19/23 (0,83) | 1066/2377 (0,45) |
| **Unknown practice type** | 2 | 0 |

In the group of non-participating GPs in the Region of Southern Denmark, male GPs and singled handed practices were overrepresented compared to the overall GP population in Denmark.

**Appendix 4:**

**5 open-ended questions and 2 closed questions prior to interviews for reflection**

1. When I hear “continuous medical education”, I think …
2. Good continuous medical education for me is …
3. When I participate in continuous medical education - I do it because …
4. If I do not participate in continuous medical education, it is due to …
5. The ideal continuous medical education for me is (dreams and visions)

Have you used your funds for accredited CME in 2018?

Have you participated in other courses (non-fundable) in 2016-2017?
